# Likelihood of a fecal occult blood test uptake among older adults: comparisons between health professionals and healthcare volunteers based on the health belief model

**DOI:** 10.1186/s12877-019-1067-5

**Published:** 2019-02-21

**Authors:** Tsung-Yi Lin, Shu-Tzu Chuang, Su-Fei Huang, Hsiao-Pei Hsu, Li-Ting Lu, Jong-Long Guo

**Affiliations:** 1 Department of Health Promotion and Health Education, National Taiwan Normal University, No.162, Sec. 1, He-ping East Road, Taipei, 10610 Taiwan; 2 Public Health Bureau, Yilan County, No.287, Sec. 2, Nuzhong Rd, Yilan City, Yilan County 26051 Taiwan; 3 Department of Senior Citizen Service, Mackay Junior College of Medicine, Nursing, and Management, No.92, Shengjing Rd., Beitou District, Taipei, 11260 Taiwan; 4 School of Nursing, National Yang-Ming University, No.155, Sec.2, Li-Nong Street, Taipei, 11221 Taiwan; 5 Department of Health Management for Elderly Society, University of Kang Ning, No.137, Alley 75, Sec. 3, Kang Ning Road, Neihu District, Taipei, 11485 Taiwan

**Keywords:** Fecal occult blood test, Health professional, Healthcare volunteer, Health belief model, Structural equation modeling, Multi-group analysis

## Abstract

**Background:**

Health professionals and healthcare volunteers play a critical role in promoting uptake of the fecal occult blood test (FOBT), which is an effective screening method for colorectal cancer. However, previous studies paid less attention to investigating both groups regarding their intention to undergo the test. This study used the Health Belief Model (HBM) to explore the likelihood of an FOBT uptake among health professionals and healthcare volunteers aged 50 years or older.

**Methods:**

A cross-sectional survey was conducted at public health centers in a county in northern Taiwan. Health professionals and healthcare volunteers were invited to complete the questionnaires. Overall, 391 valid questionnaires were obtained (response rate = 93.10%). Structural equation modeling was used to examine the associations among the variables based on the HBM.

**Results:**

The HBM explained 45, 44, and 50% of the variance in the likelihood of undergoing an FOBT in all participants, health professionals, and healthcare volunteers, respectively. The explained variance in healthcare volunteers outweighed that of professionals by 6%. Perceived benefits and self-efficacy significantly affected the likelihood of undergoing an FOBT. Self-efficacy significantly mediated the effects of perceived severity, benefits, and barriers on the likelihood of an FOBT uptake. A borderline significant difference in structural coefficients was found across groups.

**Conclusions:**

The HBM model was used to examine the likelihood of an FOBT uptake among health professionals and healthcare volunteers, and the results showed that self-efficacy was the optimal predictor of the likelihood of an FOBT uptake, followed by perceived benefits. Future multifactorial interventions to promote FOBT uptake among health professionals and healthcare volunteers aged 50–75 years could include these significant factors.

## Background

 Colorectal cancer (CRC) is the third most common cancer worldwide, and an estimated 1.36 million people are diagnosed with CRC annually [1]. In Taiwan, the incidence rate for CRC is 44.7 per 100,000 people, which is higher relative to that in other countries, and the mortality rate for CRC is 15.1 per 100,000 people [2]. Epidemiologic data have indicated that new CRC cases and deaths occur mainly in people aged 50 years or older; therefore, the recommended age range for CRC screening is 50 to 75 years. Detection of CRC at an early stage, followed by responsive treatment, can effectively reduce the incidence, morbidity, and mortality rates of CRC [3]. Indeed, biennial screening over a period of approximately 10 years reduced CRC mortality by up to 20%; even greater reductions were observed as a result of annual screening [4]. A study conducted by Chen, Lee, and Wang [5] showed that the 5-year survival rates for stages I to IV CRC ranged from 87.79 to 14.17%, respectively, and providing treatment at a younger age or an earlier cancer stage saved additional life years and healthcare costs.

In Taiwan, free CRC screening is provided through a home-based immunochemical fecal occult blood test (iFOBT) by the Department of Health for all adults aged 50 years or older [6]. The CRC screening rate in the target population is largely unsatisfactory, both in Taiwan and abroad. A U.S. survey showed that only 59% of people aged 50 years or older reported receiving CRC screening consistent with the current guidelines [7]. The screening rate in Taiwan was even lower; however, an increase in the screening rate was observed between 2010 and 2014—from 32.2 to 38.2%, respectively [8]. Therefore, it is necessary to identify the potential factors that facilitate FOBT uptake.

The Health Belief Model (HBM) is a well-known theory used to predict screening behavior and has been applied as a framework for preventive behavior programs. HBM consists of perceived susceptibility, perceived severity, perceived benefits, and perceived barriers, which influence and predict the likelihood that a given behavior will be performed [9]. Perceived susceptibility refers to the individual’s perception of the risk of contracting a given disease [10]. Perceived severity refers to the individual’s belief that a given disease or condition is serious [10, 11]. Perceived benefits refer to the belief regarding the advised behavior to reduce risk or seriousness of impact. Perceived barriers involve the individual’s assessment of the tangible and psychological costs of the advised behavior [9]. A meta-analysis has shown that the perceived benefits and barriers are optimal predictors of adopting a behavior, while the perceived susceptibility and severity exert relatively weak effects [12].

HBM can involve other factors, and cues to action and self-efficacy are frequently included as modifying factors [13]. Cues to action refer to strategies to activate “readiness”, to adopt the behavior, such as advertising and personal communications from health professionals, family members, or peers. Self-efficacy refers to the confidence in one’s ability to take action [14], which is a significant predictor of the likelihood of an individual performing a particular preventive behavior and enhances the applicability of HBM to the challenges involved in changing behavior [15].

Over the past two decades, researchers have applied the HBM and its extended model to discuss the factors predicting the likelihood of an individual taking a CRC screening test; however, few studies have focused on predicting the preventive behavior of health professionals working in local health departments. Health professionals play a vital role as health gatekeepers, and they are responsible for providing preventive health services to community residents, but their health beliefs might not be superior to those of the public. This could also be true for healthcare volunteers who encourage members of the public to receive cancer screening; they may not adhere to the guidelines regarding CRC screening.

We proposed that cues to action, self-efficacy, and the four beliefs in the HBM would exert indirect and direct effects on the likelihood of an FOBT uptake among health professionals and healthcare volunteers. The aims of the study were (1) to examine the direct and indirect effects of perceived susceptibility, perceived severity, perceived benefits, perceived barriers, self-efficacy, and cues to action on the likelihood of an FOBT uptake, and (2) to compare the similarities and differences in these effects between health professionals and healthcare volunteers.

## Methods

### Participants and procedure

A cross-sectional survey was conducted with health professionals and healthcare volunteers at public health centers in a county of northern Taiwan. Health professionals included physicians, dentists, nurses, pharmacists, medical technologists, and public health administrators. Healthcare volunteers included voluntary workers recruited by public health centers to assist in the provision of cancer screening. The inclusion criteria were (a) aged 50–75 years, (b) the ability to complete the questionnaire, and (c) the ability to provide written informed consent. The exclusion criterion was to receive an FOBT within the preceding 2 years. In total, 420 participants met the inclusion criteria, and 391 provided written informed consent and completed the questionnaire after three reminder phone calls. The response rate was 93.10%. The Institutional Review Board of Camillians Saint Mary’s Hospital approved this study (IRB104003).

### Measures

The questionnaire included background information, CRC risk factors [16], and subscales of the HBM. Background information included gender, age, educational level, occupation, living conditions, and marital status. The CRC risk factors included a personal history of colorectal polyps, a family history of CRC in first-degree relatives, being overweight (i.e., body mass index (BMI) > 27.0 kg/m^2^), and smoking behavior.

The HBM subscales, including perceived susceptibility, perceived severity, perceived benefits, perceived barriers, cues to action, self-efficacy, and the likelihood of an FOBT uptake, were modified in accordance with previous studies [17, 18], with the permission of the researchers who originally developed them [17, 18]. The numbers of items, values of Cronbach’s α, factor loadings, and explained variance for all variables included in the exploratory factor analysis are shown in Table 1. All HBM subscales were measured using a five-point Likert-type scale that indicated the extent that participants agreed with statements in the questionnaire, ranging from 1 (incompletely agree) to 5 (completely agree). Perceived susceptibility was measured using three items and the sample item was “I will get CRC during my lifetime.” Perceived severity referred to the severity of CRC and its potential consequences and was measured using items such as “CRC could increase my financial burden.” Perceived benefits referred to participants’ assessment of the advantages or efficacy of the FOBT in reducing CRC risk, and they were measured using items such as “The FOBT can detect early CRC.” Perceived barriers that referred to participants’ assessment of the obstacles to undertaking the FOBT were measured using items such as “I am afraid of finding out that I have CRC.”

**Table 1:** Reliability and factor loading for each variable

Variable	Items	Cronbach’s α	Factor loading	Explained variance (%)
Beliefs				
Perceived susceptibility	3	.90	.87–.93	82.90
Perceived severity	4	.86	.83–.87	71.31
Perceived benefits	3	.94	.94–.96	90.00
Perceived barriers	4	.89	.84–.91	75.23
Mediators				
Cues to action	3	.90	.89–.94	83.56
Self-efficacy	3	.94	.94–.96	89.21
Dependent variable				
Likelihood of FOBT uptake	3	.91	.89–.95	85.44

*FOBT* fecal occult blood test

Cues to action referred to the cues that promoted FOBT uptake and were measured using items such as “I will undergo an FOBT based on the suggestions of family members.” Self-efficacy referred to the degree that participants believed that they could undergo an FOBT and was measured using items such as “I have the ability to take time to undergo an FOBT.” The likelihood of FOBT uptake referred to the probability of undergoing an FOBT in the near future and was assessed using items such as “I plan to undergo an FOBT within the next 6 months.”

### Data analyses

IBM SPSS version 22.0 was used to perform the descriptive analysis of sociodemographic data and between-group comparisons, as well as to calculate Pearson’s correlation coefficients for the associations between factors. Structural equation modeling was performed using IBM SPSS AMOS version 23. In accordance with the two-stage approach suggested by Anderson and Gerbing [19], we initially assessed the fit of the measurement model by a confirmatory factor analysis. This analysis assessed the associations between the latent variables and factors, to support the subsequent assessment of the structural model. The structural model was then assessed, as the measurement model showed good fit. Standardized coefficient estimates with bias-corrected 95% confidence intervals were calculated using bootstrapping analysis with 1000 bootstrap samples [20].

The fit of the measurement and structural models was assessed using the following goodness-of-fit indices: comparative fit index (CFI) > .90, root mean square error of approximation (RMSEA) < .08, standardized root mean square residual (SRMR) < .08, and Tucker-Lewis index (TLI) < .90. These thresholds were based on the recommendations of Hooper, Coughlan, and Mullen [21] and Kline [22]. Because χ^2^ is sensitive to large sample sizes, some researchers have suggested that it is inappropriately used to determine model fit [21].

To assess differences in the structural coefficients of the hypothesized models between health professionals and healthcare volunteers, multi-group analyses were performed by a sequence of planned, nested comparisons of models with appropriate equality constraints on the parameters [23]. The first model was unconstrained as a baseline model. The second model assumed factor loadings constrained equal, while others were not constrained. The third model added constraints on the correlations (covariance) in addition to model two. The fourth model added constraints on the error terms (variance) in addition to model three. The fifth model additionally imposed all structural coefficients (regression weights) equal constrained between latent variables based on the model four. It was evident that at least one of the structural coefficients differed across groups as invariance tests were significant for models four and five. Consequently, this study examined each structural coefficient in turn by constraining the specific one and comparing with the fourth model. The change in χ^2^ values was used to determine the significance.

## Results

### Background information

A comparison of health professionals’ and healthcare volunteers’ background information is shown in Table 2. Gender (χ^2^ = 5.79, *p* = .016), age group (χ^2^ = 60.92, *p* < .001), educational level (χ^2^ = 62.11, *p* < .001), and occupation (χ^2^ = 173.79, *p* < .001) differed significantly between groups. That is, the proportions of men, individuals aged 50–59 years, participants with a college education or above, and participants with full-time employment were higher among health professionals than among healthcare volunteers.

**Table 2:** Comparison of background information between health professionals and healthcare volunteers

Characteristics	All (*N* = 391)	Health professionals (*N* = 191)	Healthcare volunteers (*N* = 200)	χ^2^	*p*
	n (%)	n (%)	n (%)		
Gender				5.79	.016
Male	75 (19.20)	46 (24.08)	29 (14.50)		
Female	316 (80.80)	145 (75.92)	171 (85.50)		
Age group (years)				60.92	< .001
50–59	259 (66.20)	163 (85.43)	96 (48.00)		
≥ 60	132 (33.80)	28 (14.66)	104 (52.00)		
Educational level ^a^				62.11	< .001
Year 12 or below	249 (64.62)	84 (44.92)	165 (83.33)		
College or above	136 (35.32)	103 (55.08)	33 (16.67)		
Occupation ^a^				173.79	< .001
Full-time employment	199 (51.00)	162 (85.30)	37 (18.50)		
Other	191 (49.00)	28 (14.80)	163 (81.50)		
Living conditions ^a^				2.79	.095
With family	362 (93.10)	181 (95.30)	181 (91.00)		
Other	27 (6.90)	9 (4.70)	18 (9.00)		
Marital status ^a^				3.21	.073
Married	364 (93.80)	174 (91.60)	190 (96.00)		
Other	24 (6.20)	16 (8.40)	8 (4.00)		
Colorectal polyps				1.51	.219
Yes	21 (5.40)	13 (6.80)	8 (4.00)		
No	370 (94.60)	178 (93.20)	192 (96.00)		
Family history of CRC (first-degree relative) ^a^				.05	.817
Yes	32 (8.30)	15 (7.90)	17 (8.60)		
No	355 (91.70)	174 (92.10)	181 (91.40)		
BMI (> 27 kg/m^2^) ^a^				.59	.441
Overweight	76 (19.90)	34 (18.30)	42 (21.40)		
Normal	306 (80.10)	152 (81.70)	154 (78.60)		
Smoking				.01	.911
Current or ex-smoker	22 (5.60)	11 (5.80)	11 (5.50)		
Non-smoker	369 (94.40)	180 (94.20)	189 (94.50)		

*Abbreviations*: *BMI* body mass index, *CRC* colorectal cancer

^a^missing

**Table 3:** Pearson’s correlation matrix for seven variables

	1	2	3	4	5	6	7
1. Perceived susceptibility	1	.09	.03	.14^**^	.02	- .05	.00
2. Perceived severity		1	.34^**^	- .16^**^	.21^**^	.26^**^	.28^**^
3. Perceived benefits			1	- .28^**^	.27^**^	.33^**^	.41^**^
4. Perceived barriers				1	- .37^**^	- .39^**^	- .31^**^
5. Cues to action					1	.44^**^	.27^**^
6. Self-efficacy						1	.60^**^
7. Likelihood of an FOBT uptake							1

*Abbreviations*: *FOBT* fecal occult blood test

^*^p < .05, ^**^p < .01

### Measurement model of latent constructs

**Fig. 1:**
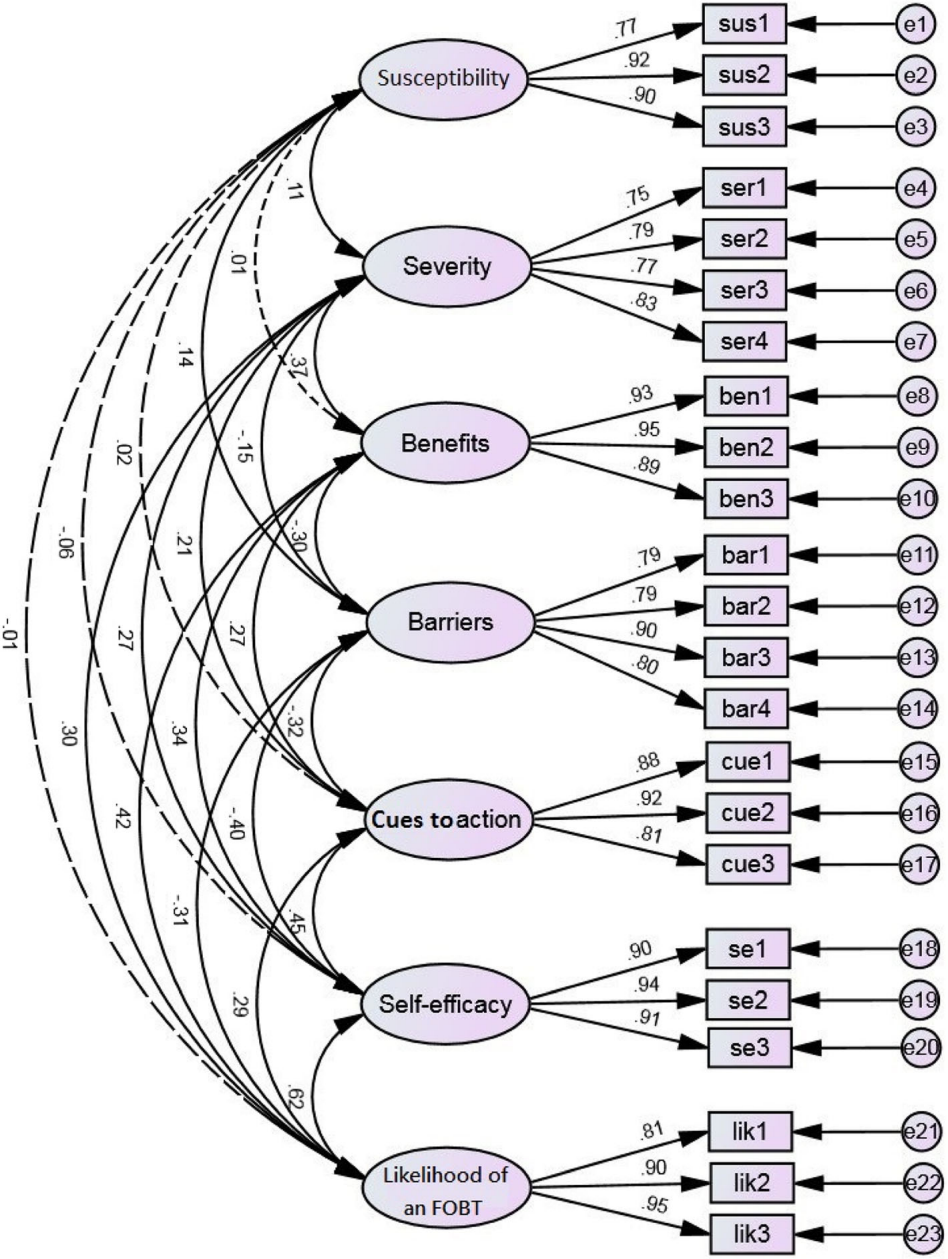
Measurement model; sus = susceptibility; ser = severity; ben = benefits; bar = barriers; se = self-efficacy; cue = cues to action; lik = likelihood

Pearson’s correlation analysis revealed that perceived severity, perceived benefits, perceived barriers, cues to action, and self-efficacy were all associated with the likelihood of an FOBT uptake (Table 3). Then, the measurement model was tested by estimating the association of each item with its hypothesized latent construct (Fig. 1). The measurement model exhibited an adequate fit to the data (χ^2^ = 456.11, *p* < .001; CFI = .96; RMSEA = .06; SRMR = .04; TLI = .96) among all participants. This study also tested the models for health professionals and healthcare volunteers separately. Both had acceptable model fit (health professionals/health volunteers: χ^2^ = 376.52/426.38, *p* < .001 for both; CFI = .95/.94; RMSEA = .07/.07; SRMR = .05/.05; TLI = .94/.93).

### Structural equation model

Based on the hypothesized relationships between constructs, the structural models for all participants and for the health professionals and healthcare volunteers were assessed individually (Tables 4 and 5). The model showed a satisfactory fit to the data for all participants (χ^2^ = 479.55, *p* < .001; CFI = .96; RMSEA = .05; SRMR = .04; TLI = .96) and accounted for 45% of the variance in the likelihood of an FOBT uptake. Half the paths were supported, and half were not supported. The standardized direct effects of self-efficacy (β = .53, *p* < .01) and perceived benefits (β = .21, *p* < .01) on the likelihood of an FOBT uptake were significant. When the indirect effects were added to the direct effects in the model, perceived severity (β = .08, *p* < .05) and perceived barriers (β = −.16, *p* < .01) also exerted significant effects on the likelihood of an FOBT uptake.

**Table 4:** Standardized structural coefficients of structured models

Paths	All (R^2^ = .45, 95% CI = .33–.55)				Health professionals (R^2^ = .44, 95% CI = .26–.58)				Healthcare volunteers (R^2^ = .50, 95% CI = .30–.66)			
	β	L	U	Result	β	L	U	Result	β	L	U	Result
Susceptibility → Likelihood of an FOBT uptake	.02	−.08	.10	NS	.01	−.10	.13	NS	.01	−.12	.15	NS
Severity → Likelihood of an FOBT uptake	.08	−.02	.20	NS	.14	−.04	.34	NS	.03	−.11	.17	NS
Benefits → Likelihood of an FOBT uptake	.21^**^	.10	.36	S	.21^**^	.06	.41	S	.17^*^	.02	.37	S
Barriers → Likelihood of an FOBT uptake	−.03	−.15	.08	NS	−.13^*^	−.29	−.01	S	.07	−.10	.27	NS
Cues to action → Likelihood of an FOBT uptake	−.04	−.17	.09	NS	−.06	−.29	.11	NS	−.01	−.19	.16	NS
Self-efficacy → Likelihood of an FOBT uptake	.53^**^	.35	.69	S	.44^**^	.24	.69	S	.66^**^	.41	.83	S
Susceptibility → Cues to action	.05	−.05	.15	NS	.05	−.13	.21	NS	.06	−.08	.22	NS
Severity → Cues to action	.11	−.04	.25	NS	.14	−.05	.33	NS	.09	−.09	.33	NS
Benefits → Cues to action	.15^*^	.03	.29	S	.08	−.11	.30	NS	.25^**^	.11	.41	S
Barriers → Cues to action	−.26^**^	−.41	−.12	S	−.30^**^	−.51	−.10	S	−.23^*^	−.42	−.04	S
Susceptibility → Self-efficacy	−.04	−.13	.06	NS	.00	−.16	.14	NS	−.05	−.18	.10	NS
Severity → Self-efficacy	.15^*^	.02	.32	S	.21^*^	.03	.41	S	.07	−.09	.28	NS
Benefits → Self-efficacy	.19^**^	.06	.34	S	.23^**^	.07	.45	S	.18^*^	.01	.36	S
Barriers → Self-efficacy	−.32^**^	−.46	−.19	S	−.32^**^	−.52	−.18	S	−.33^**^	−.54	−.13	S

Gender was controlled for in the models

*Abbreviations*: *β* standardized regression weights, *CI* confidence interval, *L* lower limit of 95% CI, *U* upper limit of 95% CI, *S* supported, *NS* not supported

^*^p < .05, ^**^p < .01

**Table 5:** Standardized direct and indirect effects on the likelihood of an FOBT uptake

	All, β (95% CI)			Professionals, β (95% CI)			Volunteers, β (95% CI)		
	Direct	Indirect	Total	Direct	Indirect	Total	Direct	Indirect	Total
Susceptibility	.02 (−.08, .10)	−.02 (−.08, .03)	.00 (−.11, .10)	.01 (−.10, .13)	.00 (−.07, .06)	.01 (−.11, .14)	.01 (−.12, .15)	−.03 (−.13, .06)	−.02 (−.18, .14)
Severity	.08 (−.02, .20)	.08^*^ (.01, .18)	.16^*^ (.01, .33)	.14 (−.04, .34)	.08^*^ (.01, .20)	.22^*^ (.02, .46)	.03 (−.11, .17)	.05 (−.05, .20)	.08 (−.11, .27)
Benefits	.21^**^ (.10, .36)	.09^**^ (.03, .18)	.30^**^ (.16, .48)	.21^**^ (.06, .41)	.10^**^ (.03, .24)	.31^**^ (.11, .54)	.17^*^ (.03, .37)	.12^*^ (.01, .24)	.29^**^ (.13, .51)
Barriers	−.03 (−.15, .08)	−.16^**^ (−.27, −.09)	−.19^**^ (−.33, −.08)	−.13^*^ (−.29, −.01)	−.12^**^ (−.27, −.04)	−.25^**^ (−.46, −.11)	.07 (−.10, .27)	−.22^**^ (−.40, −.11)	−.15 (−.34, .03)
Cues to action	−.04 (−.17, .09)	–	−.04 (−.17, .09)	−.06 (−.29, .11)	–	−.06 (−.29, .11)	−.01 (−.19, .16)	–	−.01 (−.19, .16)
Self-efficacy	.53^**^ (.35, .69)	–	.53^**^ (.35, .69)	.44^**^ (.24, .69)	–	.44^**^ (.24, .69)	.66^**^ (.41, .83)	–	.66^**^ (.41, .83)

Gender was controlled for in the models

*Abbreviations*: *β* standardized regression weights, *CI* confidence interval

^*^p < .05, ^**^p < .01

Among health professionals, the model showed a satisfactory fit to the data (χ^2^ = 400.44, *p* < .001; CFI = .95; RMSEA = .06; SRMR = .05; TLI = .94) and accounted for 44% of the variance in the likelihood of an FOBT uptake. Seven paths were supported. The standardized direct effects of self-efficacy (β = .44, *p* < .01), perceived benefits (β = .21, *p* < .01), and perceived barriers (β = −.13, *p* < .05) on the likelihood of an FOBT uptake were significant. When the indirect effects were added to the direct effects in the model, perceived severity (β = .08, *p* < .05) also exerted a significant effect on the likelihood of an FOBT uptake.

The model showed a satisfactory fit to the data among healthcare volunteers (χ^2^ = 444.05, *p* < .001; CFI = .94; RMSEA = .07; SRMR = .05; TLI = .93), accounting for 50% of the variance in the likelihood of an FOBT uptake. Six paths were supported. The standardized direct effects of self-efficacy (β = .66, *p* < .01), and perceived benefits (β = .17, *p* < .05) on the likelihood of an FOBT uptake were both significant. When the indirect effects were added to the direct effects in the model, the findings were similar with those of the health professionals.

### Multi-group analyses

**Fig. 2:**
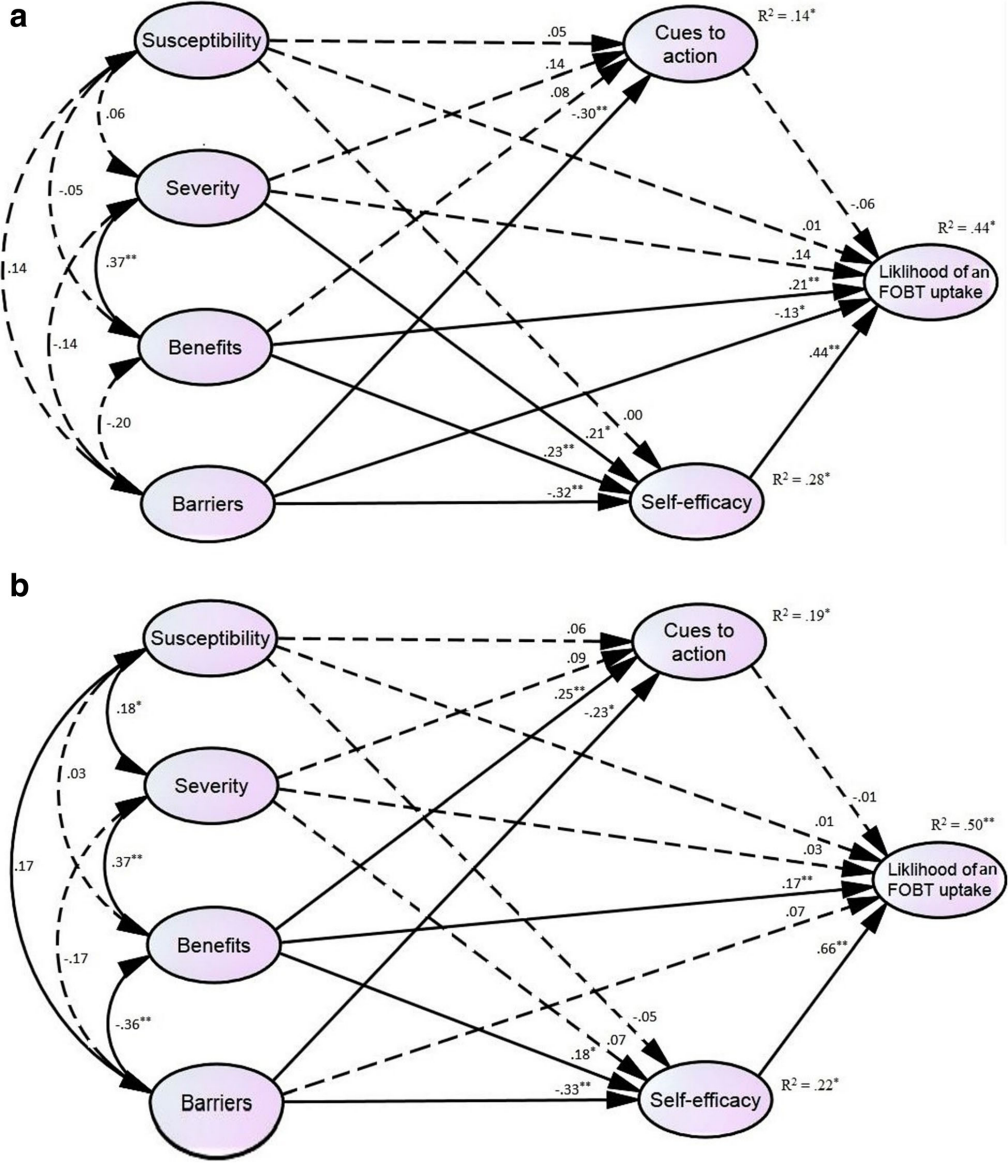
Structured models among health professionals and healthcare volunteers controlled for gender. **a** Health professionals. **b** Healthcare volunteers

As shown in Fig. 2, the structural coefficients (regression weights) differed between health professionals and healthcare volunteers. Group differences were examined using multi-group analyses (Table 6). A borderline significant difference was found between models 4 and 5 (△χ^2^_(df = 15)_ = 24.18, *p* = .062). We still examined the differences in each structural coefficient between the two groups. The paths from perceived barriers (△χ^2^_(df = 1)_ = 4.34, *p* = .037) and self-efficacy (△χ^2^_(df = 1)_ = 8.57, *p* = .003) to the likelihood of FOBT uptake significantly differed between groups.

**Table 6:** Comparisons of nested models with constrained parameters

Model		χ^2^	df	CFI	Nested models	△χ^2^	△df	p
1	Baseline: unconstrained	844.49	454	.95				
2	Factor loadings constrained equal	860.54	466	.95	2–1	16.06	12	.189
3	Factor loadings, factor correlations constrained equal	879.45	479	.94	3–2	18.90	13	.126
4	Factor loading, factor correlations, measurement error constrained equal	896.61	495	.94	4–3	17.17	16	.375
5	Factor loading, factor correlations, measurement error, structural coefficients constrained equal	920.79	510	.94	5–4	24.18	15	.062
6a	Susceptibility → Likelihood of an FOBT uptake	896.61	496	.94	6a-4	.00	1	.967
6b	Severity → Likelihood of an FOBT uptake	897.82	496	.94	6b-4	1.20	1	.272
6c	Benefits → Likelihood of an FOBT uptake	896.61	496	.94	6c-4	.00	1	.988
6d	Barriers → Likelihood of an FOBT uptake	900.95	496	.94	6d-4	4.34	1	.037
6e	Cues to action → Likelihood of an FOBT uptake	896.75	496	.94	6e-4	.14	1	.713
6f	Self-efficacy → Likelihood of an FOBT uptake	905.18	496	.94	6f-4	8.57	1	.003
6 g	Susceptibility → Cues to action	896.61	496	.94	6 g-4	.00	1	.960
6 h	Severity → Cues to action	896.73	496	.94	6 h-4	.12	1	.733
6i	Benefits → Cues to action	900.04	496	.94	6i-4	3.43	1	.064
6j	Barriers → Cues to action	898.26	496	.94	6j-4	1.65	1	.199
6 k	Susceptibility → Self-efficacy	896.80	496	.94	6 k-4	.19	1	.664
6 l	Severity → Self-efficacy	898.31	496	.94	6 l-4	1.70	1	.192
6 m	Benefits → Self-efficacy	896.81	496	.94	6 m-4	.20	1	.654
6n	Barriers → Self-efficacy	898.61	496	.94	6n-4	2.00	1	.158
6o	Gender → Likelihood of an FOBT uptake	896.64	496	.94	6o-4	.03	1	.856

*Abbreviations*: *df* degree of freedom, *CFI* comparative fit index

## Discussion

Previous systematic reviews have argued that the HBM has some limitations in explaining the uptake of the screening behavior [12, 24], but this study attempted to address these limitations. First, health professionals and healthcare volunteers were recruited to address the effects of certain contextual constraints on the model. Second, the HBM is considered by some to be a “victim-blaming” theory; however, this was not an issue in this study given the specific background of the participants in providing health services to community residents. Third, the outcome variable was the likelihood of future FOBT uptake in this study; therefore, the differences between first-time and repeated screening behavior were not considered. This study developed a questionnaire with satisfactory validity and reliability with added self-efficacy to enhance the model. A structural equation model was used to assess the associations between the HBM variables, and the multi-group analyses were performed to examine group differences.

The HBM variables explained 45, 44, and 50% of the variance in the likelihood of FOBT uptake among all participants, health professionals, and healthcare volunteers respectively. These proportions are higher than the 36% of the variance in the intention to pursue genetic tests for CRC explained in a previous study [25]. They are also higher or similar to the variances explained in previous research on the HBM, with accounting for 25% of the variance in young people’s intention to seek mental health help [26], 42.4% of the variance in brushing behavior [27], and 50.5% of the variance in antihypertensive medication adherence [28]. The current results suggest that integrating self-efficacy with the HBM increased the explained variance in the likelihood of screening behaviors. When health professionals and healthcare volunteers were examined separately, the variance in the likelihood of an FOBT uptake explained in healthcare volunteers outweighed that of health professionals by 6%, mainly because of the high path coefficients (β = .66, *p* < .01) observed between self-efficacy and the likelihood of an FOBT uptake.

Table 7 shows a comparison of the results of this study with those in previous similar studies. Self-efficacy exerted the strongest direct influence on the likelihood of FOBT uptake, which was consistent with a previous study about CRC screening [29]. Self-efficacy mediated the likelihood of FOBT uptake via three health beliefs: perceived severity, benefits, and barriers.

**Table 7:** Comparison of findings between this study and past literature regarding HBM-related predictors of CRC screening

Author (year)	Type of participants	Dependent variable	Significant HBM-related factors
The present study (2019)	Health professionals aged 50–75 years	Likelihood of FOBT uptake	Self-efficacy, perceived severity, benefits, and barriers
	Healthcare volunteers aged 50–75 years	Likelihood of FOBT uptake	Self-efficacy, and perceived benefits.
Sohler et al. (2015) [29]	Patients aged 50–75 years	Uptake of CRC screening (medical record review)	Self-efficacy, and cues to action (discussion of screening with healthcare provider)
Wong et al. (2013) [18]	Residents aged ≥50 years	Uptake of CRC screening (Colonoscopy)	Cues to action (physician’s recommendation), perceived susceptibility, and perceived barriers
Cyr et al. (2010) [25]	Residents (91.3% ≥36 years)	Intention to undergo genetic testing for CRC	Perceived benefits and barriers
Sung et al. (2008) [30]	Residents aged 30–65 years	Uptake of CRC testing	Cues to action (physician’s recommendation), perceived severity^a^, and perceived barriers
Manne et al. (2003) [31]	Siblings (aged ≥35 years) of individuals with CRC	Colonoscopy Intentions	Perceived severity, benefits, and barriers
Codori et al. (2001) [32]	First-degree relatives of patients with CRC aged 18–86 years	Past CRC Endoscopic Screening	Perceived susceptibility

^a^Perceived severity was negatively associated with the uptake of CRC testing

The finding that cues to action did not significantly predict the likelihood of FOBT uptake was inconsistent with other previous studies [18, 29, 30], indicating that cues to action (e.g., discussion of screening with a provider or physician recommendations) were relatively significant for patients and the general population. Because our participants were all involved in health services with community residents or patients, it is unsurprising that cues to action were less relevant.

The finding that perceived benefits had a direct positive effect on the likelihood of an FOBT uptake in both groups was consistent with prior findings [25, 31]. Similarly, the finding that perceived barriers had a significant negative effect on the likelihood of an FOBT uptake among health professionals was consistent with prior findings among the general population [18, 25, 30, 31].

In line with a previous study [31], perceived severity had an indirect positive effect on the likelihood of an FOBT uptake via self-efficacy. This finding is inconsistent with a previous study reporting a negative association between perceived severity and uptake of screening tests [30]. Owing to the participants’ background as healthcare practitioners, they were likely to have a positive perception of CRC screening.

It was not surprising to find that perceived susceptibility did not exert direct or indirect effects on the likelihood of FOBT uptake for either group. This was inconsistent with a previous study among first-degree relatives of patients with CRC [32], in which individuals who believed that they were highly likely to develop CRC were 1.7 times as likely to have undergone screening relative to those who believed that they were somewhat unlikely or very unlikely to develop CRC (*p* = .03). The difference implies that our participants subjectively assess themselves at the low risk of developing CRC.

Group differences were found in the paths from perceived barriers and self-efficacy to the likelihood of an FOBT uptake; however, a borderline significant trend was found across groups. The findings suggests that perceived barriers influenced the likelihood among health professionals, but not among healthcare volunteers. The effect of self-efficacy on the likelihood of an FOBT uptake was more robust in healthcare volunteers than in health professionals. These factors would be considered when developing effective programs aimed at promoting an FOBT uptake among these groups.

Based on our findings, the HBM with self-efficacy was a satisfactory model for explaining the likelihood of FOBT uptake; however, some clinical concerns by the U.S. Preventive Services Task Force need considering when promoting iFOBT, even though iFOBT is associated with reduced CRC deaths [33, 34]. The first concern is the age range for the uptake of an FOBT. Undergoing an FOBT is cost-effective for adults aged 50–75 years, but the decision to screen for CRC in adults over 75-years-old is appropriately made on an individual basis, considering the individual’s physical health and prior screening history. Similarly, an individual determined to have a high risk of developing CRC is vital to be assessed by a physicians on the timing of undertaking an FOBT. A prior study suggested patients with a family history of CRC, e.g., a first-degree relative with early-onset CRC, to undergo a colonoscopy at a younger age [35].

The HBM can be used as a framework to develop effective intervention programs for CRC screening. Previous studies have indicated that interventions applying health beliefs demonstrated favorable effects on other types of cancer screening [36, 37] and osteoporosis prevention [38]. Similarly, CRC screening could also be improved by providing HBM-based intervention program, particularly for the FOBT, which is easier to promote compared with colorectal endoscopy.

This study had some limitations. First, health professionals and healthcare volunteers might be more inclined to engage in health protective behaviors compared to the general population. Thus, caution need to be used when generalizing our findings to the general population requires caution. Second, this study established associations between the four health beliefs, cues to action, self-efficacy, and the likelihood of FOBT uptake; however, we did not examine the CRC screening behavior because the current purpose was not to identify difference between first time and repeated screening behavior. Future studies could identify suitable strategies for solving this problem. Third, those in the general population with an increased risk of CRC could be included in future research to make more insightful comparisons.

## Conclusion

The HBM model was used to examine the likelihood of FOBT uptake among health professionals and healthcare volunteers. The results showed that self-efficacy was the optimal predictor of the likelihood of an FOBT uptake, followed by perceived benefits. For health professionals and healthcare volunteers aged 50–75 years, the development of future multifactorial interventions to promote an FOBT uptake could include these HBM factors.

## Abbreviations

BMI: Body mass index
CFI: Comparative fit index
CRC: Colorectal cancer
FOBT: Fecal occult blood test
HBM: Health belief model
RMSEA: Root mean square error of approximation
SRMR: Standardized root mean square residual
TLI: Tucker–Lewis index
